# Association of endothelial glycocalyx shedding and coronary microcirculation assessed by an angiography-derived index of microcirculatory resistance in patients with suspected coronary artery disease

**DOI:** 10.3389/fcvm.2022.950102

**Published:** 2022-09-08

**Authors:** Yang Liu, Si Chen, Shaoyan Liu, Guoqiang Sun, Zhijun Sun, Hongbin Liu

**Affiliations:** ^1^Medical School of Chinese People’s Liberation Army, Beijing, China; ^2^Department of Cardiology, Second Medical Center of Chinese People’s Liberation Army General Hospital, Beijing, China; ^3^National Clinical Research Center for Geriatric Diseases, Beijing, China; ^4^Department of Cardiology, First Medical Center of Chinese People’s Liberation Army General Hospital, Beijing, China; ^5^Department of Cardiology, Yantai Municipal Laiyang Central Hospital, Yantai, China; ^6^Department of Cardiology, Sixth Medical Center of Chinese People’s Liberation Army General Hospital, Beijing, China; ^7^Beijing Key Laboratory of Chronic Heart Failure Precision Medicine, Beijing, China

**Keywords:** endothelial glycocalyx, syndecan-1, coronary microcirculation, coronary microvascular dysfunction, impaired microvascular vasodilatory capacity, angiography-derived index of microcirculatory resistance

## Abstract

**Background:**

The endothelial glycocalyx (EG) is essential for maintaining microvascular homeostasis. However, the relationship between the EG and coronary microcirculation remains to be elucidated. One of the main components of EG is syndecan-1, and its shedding has been claimed to represent the state of the EG. In this study, we aimed to analyze the association between syndecan-1 and the coronary microcirculation.

**Methods:**

We enrolled suspected coronary artery disease (CAD) patients who consecutively underwent coronary angiography (CAG) and angiography-based analysis of physiological indices in the left anterior descending artery (LAD). Serum syndecan-1 was measured by enzyme-linked immunosorbent assay (ELISA). The coronary microcirculation was evaluated by the presence of coronary microvascular dysfunction (CMD) and an impaired microvascular vasodilatory capacity (IMVC), which were quantified by an angiography-derived index of microcirculatory resistance (IMRangio) in the maximum hyperemic state (H-IMRangio) induced by adenosine triphosphate and the ratio (RRRangio) of IMRangio in the non-hyperemic phase to H-IMRangio, respectively.

**Results:**

A total of 528 patients were enrolled in this study. There was no difference in epicardial coronary complexity between patients with high syndecan-1 (HSG) and low syndecan-1 (LSG) levels grouped by the median concentration of syndecan-1 (SYNTAX: 7[3, 10] vs. 9[4, 12], *P* = 0.15). However, H-IMRangio and RRRangio were different between the LSG and HSG groups (H-IMRangio: 23.64 ± 6.28 vs. 27.67 ± 5.59, *P* < 0.01; RRRangio: 1.74[1.46, 2.08] vs. 1.55[1.34, 1.72], *P* < 0.01). Patients with CMD (H-IMRangio > 25) and patients with IMVC (RRRangio below the median value) both had higher syndecan-1 levels (CMD: 86.44 ± 54.15 vs. 55.2 ± 43.72, *P* < 0.01; IMVC: 83.86 ± 55.41 vs. 59.68 ± 45.06, *P* < 0.01). After adjustment for confounding factors, HSG remained associated with the presence of CMD and IMVC (CMD: odds ratio [OR]: 2.769, *P* < 0.01; IMVC: OR: 1.908, *P* < 0.01).

**Conclusion:**

High levels of syndecan-1 are independently associated with the presence of CMD and IMVC among patients with suspected CAD.

## Introduction

The diagnosis and treatment of coronary artery disease (CAD) mostly focus on epicardial vessels. With the development of coronary interventions, the tools used to address epicardial vascular stenosis are becoming increasingly abundant and advanced. However, various studies have shown that among patients who are undergoing clinically indicated coronary angiography, up to 49% do not have significant stenosis. Of these patients, up to 60% may have coronary microvascular dysfunction (CMD) (1–3). Over the past 2 decades, understanding of the pathophysiology of CMD has increased. The incidence of major adverse cardiovascular events (MACEs) was found to be significantly higher among patients with CMD than among patients with normal endothelial function (4–8). One study found that 18, 10, and 5% of patients with severe, moderate and mild CMD, respectively, had adverse cardiovascular events (9).

Coronary microvascular functional and structural abnormalities disrupt vasodilation, thereby limiting increased coronary blood flow in response to increased myocardial oxygen demand. Endothelial function is essential for coronary microcirculation. Functionally intact endothelium, influenced by local metabolic activity, promotes relaxation that adapts to the increase in myocardial oxygen demand (10).

The endothelial glycocalyx (EG) is a polymeric sugar-rich network covering the surface of the vascular endothelium, consisting of glycosaminoglycans, glycoproteins, glycolipids, and proteoglycans, including the syndecan family. The vast majority of the glycocalyx volume is located in the microcirculation, particularly the capillaries. The EG is essential for maintaining microvascular homeostasis by modulating vascular resistance, regulating signals, and exerting a protective effect against circulating cytokines and cells, which all trigger alterations in microcirculation (11). Consequently, the EG is essential for the endothelium to be functionally intact. However, the relationship between an impaired EG and the development of CMD has not been confirmed.

The index of microvascular resistance (IMR), obtained by the fractional flow reserve (FFR) system with the temperature dilution method, is supposed to be the gold standard for the evaluation of CMD (12, 13). However, due to the high costs and operation time requirements, the application of IMR has certain limitations, especially for consecutive clinical observation and patients with mild epicardial vascular stenosis. Quantitative flow ratio (QFR) is a new technique for evaluating coronary physiological indices without a guidewire based on coronary angiography images. A novel pressure wire-free and angiography-based index of microcirculatory resistance (IMR_*angio*_) based on QFR has been demonstrated to be a viable alternative to IMR obtained by the FFR system and temperature dilution method, with the potential to significantly simplify the assessment of CMD in patients with acute and chronic coronary syndromes regardless of epicardial stenosis (14, 15). With this technique, the measurement of IMR_*angio*_ is clearly easier to carry out in a more general population.

Based on this new technology, we aimed to investigate the association between the status of the EG and coronary microcirculation by assessing serum levels of syndecan-1, a core component of the EG, and correlate the findings with those of IMR_*angio.*_

## Materials and methods

### Study design and subjects

This was a single-center, prospective study. We enrolled consecutive patients who were admitted to the Department of Cardiology of Yantai Municipal Laiyang Central Hospital for suspected CAD, which including suspected stable angina pectoris or asymptomatic myocardial ischemia detected by physiological assessment or scintigraphy. Patients with a history of coronary intervention and other heart diseases (cardiomyopathy, heart valve disease, myocarditis, congenital heart disease), hematological system disease, cancers, and hepatic or renal insufficiency as well as patients who had undergone an invasive operation or had a severe infection within the previous 3 months were excluded from the study. In addition, patients with asthma or sick sinus syndrome and atrial-ventricular block were excluded because of contraindications to adenosine triphosphate (ATP). The study was approved by the Ethics Committee of Yantai Municipal Laiyang Central Hospital, and all patients provided informed consent.

### Anthropometric and laboratory analysis

On the morning after admission, fasting venous blood samples were collected from the median cubital vein among all enrolled patients and tested for routine blood and biochemical indices. These indicators included fasting blood glucose, creatinine, total cholesterol (TC), triglycerides (TGs), low-density lipoprotein cholesterol (LDL), and high-density lipoprotein cholesterol (HDL), which were all measured with an automatic analyzer (7600P, HITACHI, Tokyo, Japan).

The serum specimens were also used for the measurement of the syndecan-1 concentration, which was performed with a commercially available immunoassay (Human Syndecan-1 ELISA Kit, Abcam, United Kingdom) according to the manufacturer’s recommendations.

### Angiographic analysis and evaluation of the coronary microcirculation

The interventional doctors at the hospital used the standard Judkins technique to perform coronary angiography and angiographic analysis. A standard dose of 200 μg of nitroglycerine was intravenously administered to each studied artery to minimize changes in coronary volume. Ioversol (50 ml: 33.9 g; Hengrui Pharmaceutical Co., Jiangsu, China), the contrast agent, was injected at a rate of 3–4 ml/s and 2–3 ml/s for the left and right coronary arteries, respectively. Images of the left and right coronary arteries were obtained from at least 2 views. All angiographies were recorded at a rate of 15 frames/s. Three experienced cardiologists were responsible for performing and analyzing the angiograms.

All enrolled patients underwent coronary angiography-based analysis of wire-free physiological indices in the left anterior descending artery (LAD) by a validated system, AngioPlus (Pulse Medical Imaging Technology, Shanghai, China) (16). The coronary angiography images were exported in DICOM format and transferred to the AngioPlus system in the Department of Cardiology of the Chinese PLA General Hospital. Two angiographic images from different angles (≥ 25°) with minimal vessel overlap were acquired. The proximal ends were located at the most proximal segment of the imaged vessel. The distal ends were located at the most distal anatomical landmarks, such as side branches. The software reconstructed a 3D anatomical vessel model without its side branches for the computation of QFR. The algorithms used for QFR assessment were described previously (16). Brief description as follows. The reconstructed vessel segment was automatically divided into several subsegments along the arterial centreline, with each three consecutive centreline points forming a subsegment. Typically, a subsegment has a length of 6 mm. The pressure drop (ΔP) for each subsegment was calculated using the stenosis geometry and contrast-flow velocity (CFV), which can be formulated as follows:


$$\Delta \,P=c\,1\times C\,F\,V+c\,2\times C\,F\,V^{\wedge }\,2$$

Where c1 and c2 are viscosity and expansion loss coefficients that were determined by the stenosis geometry characterized by the diseased lumen sizing with respect to the reference sizing. The computation of CFV was as follow. According to the length of the centerline, the length of the vessel was calculated. Considering the frame rate, the curve of vessel length variation over time (length/time curve) could be derived. The flow velocity could then be easily calculated by fitting a straight line to the length/time curve during the phase of contrast injection, using the least-square method. The slope of this fitting line defined the rate of length change over time, and hence the flow velocity.

ΔP at every position with respect to the most proximal position is calculated by integrating the pressure drop of all subsegments proximal to that interrogated location. Thus, the QFR at the interrogated position can be derived by the following formula:


$$Q\,F\,R=\frac{P\,a-\int \Delta \,P\,dx}{P\,a}$$

Where Pa is the aorta pressure and x represents the distance from the most proximal position to the interrogated position.

The evaluation of coronary microcirculation was performed according to IMR_*angio*_, which was derived from the calculation formula of IMR referred to the study of De Maria, G. L. et which is calculated as follows (15, 17):


$$I\,M\,R_{a\,n\,g\,i\,o}=P\,a\times Q\,F\,R\times \frac{N\,f\,r\,a\,m\,e\,s}{f\,p\,s}$$

**FIGURE 1:**
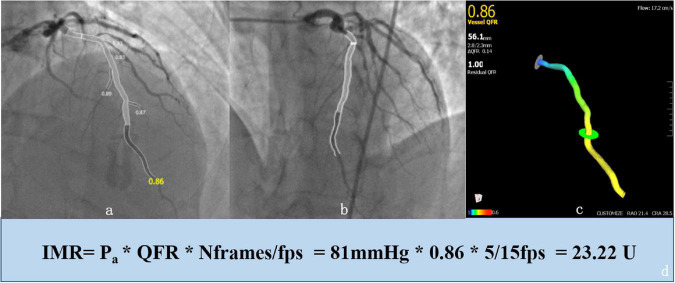
Example of an enrolled 65-year-old patient who was analyzed with the AngioPlus System to obtain the value of the quantitative flow ratio (QFR) and derivation of the angiography-based index of microcirculatory resistance (IMRangio). First, we obtained a 3D anatomical vessel model without its side branches **(c)** for the computation of the QFR by two angiographic images with different angles (≥ 25°) and minimal vessel overlap **(a,b)**. Second, the value of IMRangio was derived according to the mean baseline aortic pressure (Pa), the number of frames for contrast dye traveling from the tip of the guiding catheter to the distal reference (Nframes) and the value of the QFR **(d)**.

Where Pa is the mean baseline aortic pressure; Nframes is the number of frames for contrast dye traveling from the tip of the guide catheter to the distal reference; and fps is the frame rate (prespecified as 15). Figure 1 shows the 3D anatomical vessel model reconstruction and its automatic analyses of QFR in the LAD, which was carried out by the AngioPlus system and the derived calculation of IMR_*angio*_. With reference to the optimal cut-off values determined by previous studies, coronary microvascular dysfunction (CMD) in this study was defined as an H-IMR_*angio*_ value greater than 25 (15).

Physiological indices were obtained in the non-hyperemic and maximum hyperemic states. A maximal hyperemic state was achieved with intravenous infusion of ATP at a rate of 140 μg/kg/min. The angiography-based resistive reserve ratio (RRR_*angio*_) was calculated for enrolled patients as the ratio of IMR_*angio*_ in the non-hyperemic state (NH- IMR_*angio*_) and the maximum hyperemic state (H-IMR_*angio*_) as follows:


$$R\,R\,R_{a\,n\,g\,i\,o}=\frac{N\,H-I\,M\,R_{a\,n\,g\,i\,o}}{H-I\,M\,R_{a\,n\,g\,i\,o}}$$

RRRangio is a measurement of coronary microvascular vasodilatory capacity, describing the ability of the microcirculation to respond to a vasodilatory stimulus regardless of epicardial stenosis (15, 18, 19). Due to the lack of reference on the optimal cut-off value to define impaired coronary vasodilator capacity (IMVC) using RRRangio, referring to the methods of previous studies (15, 18, 19), we used RRRangio below the median value was used to define IMVC.

### Statistics

Data are expressed as frequencies and percentages for categorical variables and as the means ± standard deviations (if normally distributed) or as medians with interquartile ranges (if non-normally distributed) for continuous variables. Continuous variables were compared using Student’s *t* test or the Mann–Whitney *U* test. Variables relevant to the multivariable models were selected by their clinical significance and a threshold *P* value < 0.1 from the univariate analyses. Binary logistic regression analysis was performed to assess independent factors associated with CMD or IMVC. The area under the receiver-operating characteristic curve (AUC) was calculated to evaluate the predictive ability of syndecan-1 for CMD or IMVC. Analyses were performed using GraphPad Prism (version 5.0.1, San Diego, CA, United States). A *P* value < 0.05 was considered statistically significant.

## Results

### Enrolled patient characteristics

A total of 528 patients were consecutively enrolled in this study. The median age of the enrolled patients was 68 years, and the proportion of males was 49.2%. Using the median syndecan-1 level of the enrolled patients as the cutoff value (52.9 ng/ml), all subjects were divided into two groups: a high syndecan-1 group (HSG) and a low syndecan-1 group (LSG). The baseline characteristics are listed in Table 1. Patients in the HSG were older than those in the LSG. A higher prevalence of diabetes mellitus was observed in the HSG. Four hundred and twenty patients were enrolled in this study due to suspected angina pectoris and there was no significant difference between LSG and HSG. The other demographic factors did not differ significantly. Other than serum albumin, the two groups showed no significant differences in laboratory tests. The levels of serum albumin were higher in the HSG than in the LSG (51.06 ± 6.27 ng/ml vs. 44.38 ± 5.64 ng/ml, *P* < 0.01).

**TABLE 1:** Differences in baseline characteristics of patients grouped by the median syndecan-1 level.

Clinical characteristics	ALL (*n* = 528)	HSG (*n* = 264)	LSG (*n* = 264)	*P*
Age, y	68 (61, 76)	67 (59, 74)	70 (62, 78)	0.01
Male, n (%)	260 (49.24%)	126 (47.73%)	134 (50.76%)	0.54
Body mass index, kg/m^2^	28.65 ± 3.65	28.72 ± 0.23	28.58 ± 0.22	0.68
Diabetes mellitus, n (%)	237 (44.89%)	84 (31.82%)	153 (57.95%)	<0.01
Hypertension, n (%)	396 (75.00%)	193 (73.11%)	203 (76.89%)	0.37
Hypercholesterolemia, n (%)	200 (37.88%)	104 (39.39%)	96 (36.36%)	0.53
Current smoker, n (%)	110 (20.83%)	50 (18.94%)	60 (22.73%)	0.34
Total cholesterol, mg/dl	4.52 (3.52, 6.4)	4.64 (3.54, 6.39)	4.43 (3.52, 6.43)	0.86
HDL-C, mg/dl	1.1 (0.92, 1.29)	1.13 (0.94, 1.35)	1.08 (0.91, 1.25)	0.13
Total triglyceride, mg/dl	2.37 (1.21, 4.05)	2.33 (1.14, 4.1)	2.43 (1.32, 3.96)	0.74
LDL-C, mg/dl	2.97 ± 1.13	2.94 ± 0.07	2.99 ± 0.06	0.60
Creatinine, μmol/L	87.7 (71.46, 115.84)	87.96 (72.33, 116.87)	87.1 (68.92, 112.93)	0.40
Albumin, g/L	47.72 ± 6.83	51.06 ± 6.27	44.38 ± 5.64	<0.01
White blood cells (109/L)	7.29 (5.05, 9.26)	7.1 (5.1, 9.38)	7.36 (5.03, 9.20)	0.58
Red blood cells (109/L)	4.21 (3.45, 4.81)	4.17 (3.45, 4.78)	4.24 (3.46, 4.82)	0.86
Platelet count (109/L)	215 (143, 295)	216 (143, 292)	215 (143, 296)	0.58
Hemoglobin (g/L)	116.5 (103, 133.75)	119 (103, 134)	115 (103, 132)	0.19
Aspirin, n (%)	192 (36.36%)	95 (35.98%)	97 (36.74%)	0.93
ACEI/ARB, n (%)	238 (45.08%)	125 (47.35%)	113 (42.80%)	0.34
Beat receptor blocker, n (%)	104 (19.70%)	47 (17.80%)	57 (21.59%)	0.33
CCB, n (%)	108 (20.45%)	49 (18.56%)	59 (22.35%)	0.33
Statin, n (%)	318 (60.23%)	150 (56.82%)	168 (63.64%)	0.13
Suspected symptomatic CAD, n (%)	420 (79.55%)	202 (76.52%)	218 (82.85%)	0.08

Continuous variables are reported as the means ± standard deviations (if normally distributed) or as medians with interquartile ranges for continuous variables (if non-normally distributed); categorical variables are expressed as numbers (percentages). HSG, high syndecan-1 group; LSG, low syndecan-1 group; HDL-C, high-density lipoprotein cholesterol; LDL-C: low density lipoprotein cholesterol; ACEI/ARB, angiotensin-converting enzyme inhibitors/angiotensin receptor blockers; CCB, calcium channel blocker.

### Angiographic findings and physiological indices

Angiographic findings and physiological indices are presented in Table 2. Coronary complexity, which was reflected by the SYNTAX score, was not significantly different between the LSG and HSG (7[3, 10] vs. 9[4, 12], *P* = 0.15). There was no difference in the proportion of one-vessel disease and 3-vessel disease between the two groups (one-vessel disease: 39.8 vs. 35.6%, *P* = 0.37; 3-vessel disease: 8.3 vs. 9.47%, *P* = 0.76).

**TABLE 2:** Angiographic findings and physiological indices between the patients grouped by the median syndecan-1 level.

	HSG (*n* = 264)	LSG (*n* = 264)	*P*
**Angiographic findings**			
1-vessel disease, n (%)	94 (35.6%)	105 (39.8%)	0.37
2-vessel disease, n (%)	145 (54.9%)	137 (52%)	0.54
3-vessel disease, n (%)	25 (9.47%)	22 (8.3%)	0.76
SYNTAX score	13 (6, 18)	7 (3, 10)	< 0.01
**Physiological indices of LAD**			
QFR	0.85 (0.75, 0.91)	0.88 (0.80, 0.94)	0.02
QFR < 0.8, n (%)	83 (31.4%)	63 (23.9%)	0.06
Resting flow velocity, m/s	0.17 ± 0.1	0.19 ± 0.1	0.78
Hyperemic flow velocity, m/s	0.28 ± 0.1	0.42 ± 0.09	< 0.01
CFR	1.98 ± 0.62	2.32 ± 0.43	< 0.01
H-IMR_*angio*_	27.67 ± 5.59	23.64 ± 6.28	< 0.01
NH- IMR_*angio*_	34.78 (27.76, 41.68)	32.95 (26.06, 41.48)	0.1
RRR_*angio*_	1.55 (1.34, 1.72)	1.74 (1.46, 2.08)	< 0.01
IMVC, n (%)	160 (60.6%)	110 (41.67%)	< 0.01
CMD, n (%)	176 (66.7%)	104 (39.4%)	< 0.01

Continuous variables are reported as the means ± standard deviations (if normally distributed) or as medians with interquartile ranges for continuous variables (if non-normally distributed); categorical variables are expressed as numbers (percentages). HSG, high syndecan-1 group; LSG, low syndecan-1 group; SYNTAX scores, synergy between percutaneous coronary intervention with taxus and cardiac surgery scores; QFR, quantitative flow ratio; CFR, coronary flow reserve which computed as the ratio of hyperemic flow velocity and resting flow velocity; H-IMRangio, angiography-derived index of microcirculatory resistance in the maximum hyperemic state; NH-IMRangio, angiography-derived index of microcirculatory resistance in the non-hyperemic state; RRRangio, angiography-based resistive reserve ratio.

Physiological indices of the LAD were evaluated. A total of 146 patients had ischemic stenosis (QFR < 0.8) in the LAD, and a total of 280 patients were diagnosed with CMD. The proportion of patients with an ischemic LAD in the HSG was 23.9%, which was not different from that in the LSG (23.9 vs. 31.4%, *P* = 0.06). Among all the enrolled patients, there was no significant difference in the IMR_*angio*_ between patients with ischemic stenosis in the LAD compared and patients without ischemic stenosis (26.26 ± 6.61 vs. 25.44 ± 6.14, *P* = 0.18). The mean resting flow velocity of the two groups was not different (0.19 ± 0.1 m/s vs. 0.17 ± 0.1 m/s, *P* = 0.78). After ATP infusion, the mean flow velocities in the LSG significantly increased (0.42 ± 0.09 m/s vs. 0.28 ± 0.1 m/s, *P* < 0.01). Referring to the evaluation of coronary microcirculation, the NH-IMR_*angio*_ showed no difference between the HSG and LSG (32.95 [26.06, 41.48] vs. 34.78 [27.76, 41.68], *P* = 0.1). However, the H-IMR_*angio*_ was significantly lower in the LSG (23.64 ± 6.28 vs. 27.67 ± 5.59, *P* < 0.01). Moreover, we found that the proportion of CMD in the HSG was significantly higher than that in the LSG (66.7% vs. 39.4%, *P* < 0.01). In addition, this study found that RRR_*angio*_ was significantly higher in the LSG (1.74[1.46, 2.08] vs. 1.55[1.34, 1.72], *P* < 0.01), and the proportion of patients with IMVC in the LSG was higher than that in the HSG (60.6% vs. 41.67%, *P* < 0.01).

### Association of coronary microcirculation and the endothelial glycocalyx

**FIGURE 2:**
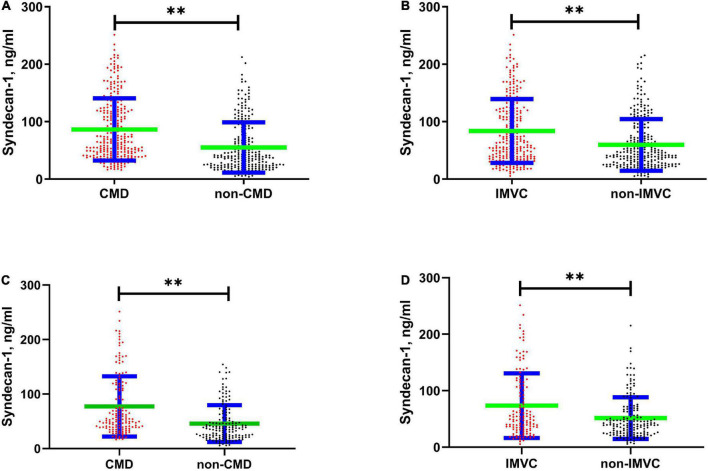
Scatter Plot showed difference in serum syndecan-1 concentration between different groups. The green horizontal line reflects the average value, and the blue horizontal line reflects the standard error. **(A)** Shows the difference between CMD and non-CMD (86.44 ± 54.15 vs. 55.2 ± 43.72, *P* < 0.01). **(B)** Shows the difference between IMVC and non-IMVC (83.86 ± 55.41 vs. 59.68 ± 45.06, *P* < 0.01). **(C)** Shows the difference between CMD and non-CMD among patients without diabetes mellites (77.36 ± 55.27 vs. 45.9 ± 33.79, *P* < 0.01). **(D)** Shows the difference between IMVC and non-IMVC among patients without diabetes mellites (IMVC vs. non-IMVC: 73.58 ± 57.23 vs. 51.51 ± 36.92, *P* < 0.01). CMD, coronary microvascular dysfunction; IMVC, impaired microvascular vasodilatory capacity. **P < 0.01.

The serum syndecan-1 concentration was significantly different when enrolled patients were grouped according to the presence of CMD and IMVC (CMD vs. non-CMD: 86.44 ± 54.15 vs. 55.2 ± 43.72 ng/ml, *P* < 0.01, Figure 2A; IMVC vs. non-IMVC: 83.86 ± 55.41 vs. 59.68 ± 45.06 ng/ml, *P* < 0.01, Figure 2B). It has been reported that syndecan-1 was significantly elevated in patients with diabetes (20, 21). Among enrolled patients, there was significant different between patients with DM and without DM (DM vs. non-DM:84.81 ± 53.51 vs. 61.14 ± 48.05 ng/ml, *P* < 0.01). To further clarify the association between EG shedding and coronary microcirculation in patients without DM, we further analyzed the difference of syndecan-1 among patients without DM (CMD vs. non-CMD: 77.36 ± 55.27 vs. 45.9 ± 33.79 ng/ml, *P* < 0.01, Figure 2C; IMVC vs. non-IMVC: 73.58 ± 57.23 vs. 51.51 ± 36.92 ng/ml, *P* < 0.01, Figure 2D). Logistic regression analysis was applied to screen out the independent factors associated with the presence of CMD and IMVC (Table 3). When variables with *P* < 0.1 in univariable regression analysis were tested in a multivariable model, high syndecan-1 levels and diabetes mellitus (DM) were found to be independently associated with the presence of CMD (high syndecan-1 level: odds ratio (OR) = 2.769, 95% confidence interval (95% CI): 1.817–4.22, *P* < 0.01; DM: OR = 1.79, 95% CI: 1.167–2.744, *P* = 0.01) and IMVC (high syndecan-1 level: OR = 1.908, 95% CI: 1.261–2.888, *P* < 0.01; DM: OR = 1.466, 95% CI: 1.018–2.112, *P* = 0.04).

**TABLE 3:** Association of coronary microvascular dysfunction and impaired microvascular vasodilatory capacity with other independent variables determined by the multivariable logistic regression models.

	Odd ratio	95% CI	SE	*P*
Factors related to coronary microvascular dysfunction. Variables selected by univariable regression analysis included high syndecan-1 level, age, male sex, current smoker, body mass index, hypertension, total cholesterol, diabetes mellitus, albumin				
High syndecan-1 level	2.769	1.817, 4.22	0.215	<0.01
Age	1.001	0.982, 1.021	0.010	0.90
Male	1.023	0.678, 1.545	0.210	0.91
Current smoker	1.262	0.754, 2.112	0.263	0.38
Body mass index	0.968	0.920, 1.018	0.026	0.21
Diabetes mellitus	1.790	1.167, 2.744	0.218	0.01
Total cholesterol	1.139	0.781, 1.661	0.193	0.50
Albumin	1.090	0.926, 1.283	0.083	0.30
Factors related to impaired microvascular vasodilatory capacity. Variables selected by univariable regression analysis included syndecan-1, age, male sex, body mass index, low-density lipoprotein cholesterol, creatinine, diabetes mellitus, albumin				
High syndecan-1 level	1.908	1.261, 2.888	0.211	<0.01
Age	1.001	0.982, 1.019	0.009	0.94
Male	0.885	0.62, 1.264	0.182	0.50
Body mass index	0.961	0.915, 1.010	0.025	0.12
low density lipoprotein cholesterol	0.93	0.793, 1.091	0.081	0.38
Creatinine	0.996	0.99, 1.002	0.003	0.15
Diabetes mellitus	1.466	1.018, 2.112	0.186	0.04
Albumin	0.098	0.952, 1.008	0.015	0.16

### Receiver operating characteristic (ROC) analysis of syndecan-1 to identify CMD and impaired microvascular vasodilatory capacity

**FIGURE 3:**
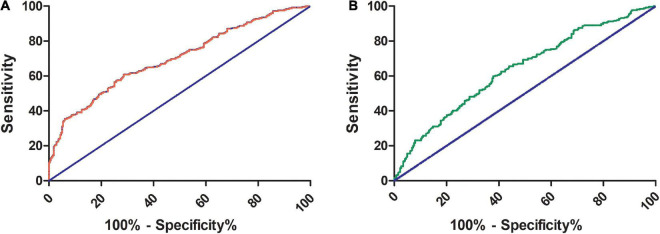
Receiver operating characteristic (ROC) curve of serum syndecan-1 for the prediction of CMD and IMVC. The areas under the ROC curve for CMD **(A)** and IMVC **(B)** were 0.7 (95% CI: 0.65–0.74, *P* < 0.01) and 0.64 (95% CI: 0.59–0.68, *P* < 0.01), respectively. For the prediction of CMD and IMVC, the cutoff values of syndecan-1 were 46.99 ng/ml (sensitivity 60.89% and specificity 71.43%) and 54.11 ng/ml (sensitivity 59.85% and specificity 62.12%), respectively. CMD, coronary microvascular dysfunction; IMVC, impaired microvascular vasodilatory capacity.

Receiver operating characteristic curves were constructed to assess the ability of the syndecan-1 value to identify CMD and IMVC. As shown in Figure 3A, the area under the curve (AUC) of syndecan-1 for CMD was 0.7 (95% CI: 0.65–0.74, *P* < 0.01), with a cutoff value of 46.99 ng/ml (sensitivity 60.89% and specificity 71.43%). As shown in Figure 3B, the AUC of syndecan-1 for IMVC was 0.64 (95% CI: 0.59–0.68, *P* < 0.01), with a cutoff value of 54.11 ng/ml (sensitivity 59.85% and specificity 62.12%).

## Discussion

To assess the relationship between the EG and coronary microcirculation, we quantitatively evaluated coronary microcirculation using an angiographic-based functional analysis of the LAD and applied the measurement of serum syndecan-1 to reflect the state of the EG. The main findings of this study are that a high serum syndecan-1 level was independently associated with the presence of CMD and an impaired microvascular vasodilatory capacity.

Recently, the important role of CMD in cardiovascular disease has been increasingly recognized. Up to 14% of patients with myocardial infarction are found to have non-obstructive coronary arteries (MINOCA) (22), which represents a diagnostic and therapeutic dilemma since many patients are discharged without a clear etiology for its clinical presentation (23, 24). As a result, patients may be treated inappropriately or not treated at all. In addition, heart failure with preserved ejection fraction (HFpEF) is viewed as the most intractable fortress in the battle against the prevention and treatment of cardiovascular disease (25). CMD was found to be closely associated with the development and progression of HFpEF. Amelioration of CMD may be a breakthrough in improving the prognosis of HFpEF (26, 27).

However, the exploration on risk factors for CMD is relatively limited. Among the recognized risk factors, female sex was once considered an important factor affecting its occurrence, except for DM. CDM related disease, such as MINOCA, is even once called “female-pattern” cardiovascular disease (28). But several real-world studies in recent years have found that men and women actually differ little in the incidence of CMD (5, 29, 30). In this study population, we similarly did not find an independent association between gender and the occurrence of CMD. Therefore, there were much work remained to be done to unearth the factors associated with CMD.

The EG is an important component in the maintenance of microcirculation (31). Syndecan-1 is a specific core component of the glycocalyx, and elevated serum concentration of syndecan-1 are highly sensitive and specific for reflecting the degree of EG disruption. However, there is still a lack of recognized normal reference range. The largest study to date on concentration of serum syndecan-1 showed that the median of serum syndecan-1 was 19.3 ng/ml (Quartile: 13.7–27.3 ng/ml), which was significantly lower than 48.81 ng/ml (Quartile: 25.34–91.19 ng/ml) in our study (32). We supposed that it may be due to aging and a relatively high proportion of complications in our enrolled patients. Another informative study on syndecan-1 in CAD patients suggested the median of serum syndecan-1 was 99 ng/ml, which was also significantly different from our data (33). According to the published research on serum syndecan-1, the fluctuation range of syndecan-1 is largely varied. The structural variability of EG is large, which ranges from 200 to 2,000 nm in thickness. The instability of its structure may be the main reason for large diversity of serum syndecan-1 (34). We expect more studies with large samples to emerge, helping us to further clarify the reference range of serum syndecan-1.

The concentration of serum syndecan-1 has been found to be significantly elevated in patients with systemic microcirculatory changes, such as sepsis, diabetes and advanced age (20, 21, 35–37). In our study, the shedding of the glycocalyx, syndecan-1, differed significantly between patients with and without diabetes among the enrolled patients and between patients grouped according to the median age, which was consistent with previous studies. However, in our cohort, there was no significant difference in the proportion of patients with symptoms between HSG and LSG. This may be related to the relatively stable condition of the enrolled patients. It has no effect on the shedding of EG by the stimulation of a state of relative no stress. In addition, asymptomatic patients were with older age and more diabetes, which promoting the shedding of EG and making up for the elevation of syndecan-1 caused by uncomfortable symptoms. Notably, a significant difference in albumin levels was found between patients grouped by syndecan-1 levels. This may be relevant to the protective function of albumin in the EG. Plasma albumin is physiologically bound within the EG, thus contributing to the stability of the layer. Preclinical studies have illustrated the mechanism of albumin and its effects in models of hemorrhagic shock, endotoxemia, vascular permeability and ischemia. The results from *in vitro* and *in vivo* experiments illustrate the multifunctional nature of albumin, including the maintenance of glycocalyx integrity, partial restoration of impaired vascular permeability and improvement of the microcirculation (38). This result provides a new perspective on strategies for EG-related diseases in the future.

The current study addressed an additional controversial question regarding whether EG shedding, syndecan-1, is associated with lesions of coronary epicardial arteries. Nemoto et al. considered EG impairment to be of little relevance to the complexity of epicardial lesions (33), while an earlier study performed by Xiangjun Xue et al. found a certain association between the shedding of the glycocalyx and the extent of coronary lesions (39). The results of the current study are similar to previous findings obtained by Nemoto et al. on this issue. However, the reasons for the different results of various studies need to be further studied. The author supposed that the different proportions of the endothelial barrier in the formation of coronary atherosclerotic plaques may determine the different strengths of the relationship between the EG and coronary lesions.

With the abundance of evaluation methods for coronary microcirculation, we have had an increasing understanding of CMD. These methods include calculation of IMR by FFR systems and evaluation by non-invasive methods such as CFR evaluated by ultrasound, Cardiac magnetic resonance, PET/CT or dynamic myocardial perfusion computer tomography. However, traditional non-invasive evaluation methods can only evaluate surrogate markers of coronary function. Moreover, contrary to obstructive CAD in which perfusion abnormalities have regional distribution, myocardial impairment in CMD may be a generalized process resulting in diffuse myocardial perfusion abnormalities (40). Therefore, non-invasive ischemia tests may be normal. CMD exists regardless of epicardial stenosis. One of the most important features of IMR in assessing coronary microcirculation is that it is not affected by the degree of epicardial vascular stenosis, which is one of the greatest advantages of IMR measurement (41, 42). Measurement of IMRangio has the both advantages invasional methods and non-invasional ones, which is regarded as a highly potential and effective way to evaluate CMD. Regarding the measurement of physiological indices in coronary arteries, we chose to conduct these in the LAD because the IMR had poor numerical agreement among different coronary arteries (43). All analyses conducted in the LAD would be more comparable among all the enrolled patients. In addition, stenosis in vessels could not affect the determination of the IMR. Consequently, measuring it in the LAD was not affected by the presence of lesions in the LAD (41). Our study similarly confirmed that the IMR of patients grouped by whether the QFR was less than 0.8 was not significantly different.

Based on analysis of the relationship between the endothelium and glycocalyx, the impact of the glycocalyx on microcirculation is two-sided (34). On the one hand, glycocalyx disruption promotes endothelial apoptosis and endothelial-to-mesenchymal transition and then affects the myocardial microvessel density (27, 44). On the other hand, the glycocalyx is not accessible for flowing red blood cells and greatly hinders plasma flow in the axial direction, causing a reduction in functionally perfused capillary volume (45). In the state of non-hyperemia, the data from our study show no association between the EG and NH-IMR_*angio*_. It is possible that the positive and negative factors that affect microcirculation resistance have reached a certain balance in long-term adaptation. However, the flow velocity in the LAD showed differences, leading to differences in H-IMR_*angio*_ and the presence of IMVC between patients grouped by syndecan-1 in the hyperemic state obtained by injection of ATP. One of the mechanisms of the ATP-induced increase in microcirculatory flow is achieved by reducing glycocalyx exclusion properties (45). Therefore, in good condition, the EG fully contributed to the expansion effect of ATP on the microcirculation. We supposed that the flow velocity difference in the state of hyperemia may be related to impairments in the EG and the resulting limited space for adenosine to exert its effects on glycocalyx exclusion property reduction.

The impact of EG impairment caused by COVID-19 on the microcirculation has been repeatedly demonstrated as the epidemic spread. EG protection has regained a focus in the domain of panvascular disease (46). Our study focuses on the association between the EG and coronary microcirculation, finding that increased EG shedding is independently associated with the presence of CMD, and the impact of the EG on CMD may mainly be achieved by decreasing microvascular vasodilatory capacity. CMD, which is independent of the occurrence of epicardial coronary artery disease, accounts for a large proportion of patients with angina. Although mature interventional techniques to relieve the stenosis of epicardial vessels, the means to prevent and relieve CMD are relatively scarce. Therapy, such as sulodexide has been attracted great attention for its protective effects on the EG (47). This study may provide a new direction for the management of patients with angina caused by CMD from the perspective of glycocalyx protection.

### Study limitations

Some limitations of our study must be noted. First, the enrolled patients had suspected CAD. The included population was relatively limited. Expanding the inclusion criteria may yield different results. Second, we did not collect data pertaining to the long-term serial coronary microvascular changes of the enrolled patients. There was no further analysis of whether syndecan-1 had an impact on the changes in coronary microcirculation. Third, limited by the inclusion criteria, we could not exclude selection bias for patients who underwent angiography, especially with a low proportion of patients with a higher stage of CKD. Fourth, the gold standard for evaluating coronary microcirculation is the IMR measured by the temperature dilution method and pressure wire. Although there was good accuracy of coronary angiography-based analysis of wire-free physiological indices in the evaluation of coronary microcirculation, there might be deviation in the results.

## Conclusion

A high level of syndecan-1, which reflects impairment of the EG, is independently associated with the presence of CMD and IMVC among patients with suspected CAD. This finding suggests an association between EG disruption and impaired coronary microcirculation. Further studies are required to demonstrate the causal relationship of the EG in the initiation and development of CMD. Furthermore, this study may provide new insight into improving the prognosis of CMD-related diseases from the perspective of EG protection.

## Data availability statement

The raw data supporting the conclusions of this article will be made available by the authors, without undue reservation.

## Ethics statement

The studies involving human participants were reviewed and approved by the Ethics Committee of Yantai Municipal Laiyang Central Hospital. The patients/participants provided their written informed consent to participate in this study. Written informed consent was obtained from the individual(s) for the publication of any potentially identifiable images or data included in this article.

## Author contributions

YL was responsible for the study design, statistical analysis, manuscript writing, and the guarantor of this work and as such had full access to all the data in the study and takes responsibility for the integrity and accuracy of the data analyses. SL was responsible for screening and informed notification of patients and performance of CAG. SC and GS were responsible for the measurement of QFR and IMRangio by AngioPlus. ZS performed the major revisions of the manuscript. HL was responsible for the conception, funding, and study design and corrected and approved the revisions and final version of the manuscript. All authors have discussed the manuscript contents.
